# Editorial Overview: Endogenous Retroviruses in Development and Disease

**DOI:** 10.3390/v12121446

**Published:** 2020-12-16

**Authors:** Molly Gale Hammell, Helen M. Rowe

**Affiliations:** 1 Cold Spring Harbor Laboratory, Cold Spring Harbor, NY 11724, USA; 2 Centre for Immunobiology, Blizard Institute, Queen Mary University of London, 4 Newark St, London E1 2AT, UK

As guest editors, we are pleased to present this Special Issue on endogenous retroviruses (ERVs) and their impact on mammalian development and disease. The breadth of articles and reviews in this issue provides valuable insight into how ERVs have shaped mammalian genomes, are regulated during development and how a proportion of them have been linked to disease. Briefly, endogenous retroviruses (ERVs) form approximately 8% of the human genome, and derive from ancient retroviral insertions into the genomes of our distant ancestors. Most human ERV sequences exist as fragments and/or mutated versions of the proviral sequences that initially integrated, with no known full length HERV proviruses able to act as replication competent retroviruses in the human genome. Despite this, many human ERV sequences retain some level of function that may be either neutral, detrimental or, in some cases, beneficial to the host. In contrast, several mouse ERV families are still active and can create new genomic copies of themselves in addition to serving as regulatory elements and contributing to other detrimental and/or beneficial interactions with the host. Each of the primary research articles in this special issue tackles a fundamental question in ERV biology, from identifying new host control factors to showing new evidence for disease associations. The review articles cover a wide range of topics, providing an overview of the state of ERV biology and outlining the many open questions that remain.

One reason that ERVs remain underexplored in mammalian development and disease is the difficulty in studying these sequences using traditional genetics and genomics approaches. These highly repetitive sequences can be present at hundreds of loci in the genome and some of these loci differ by only a few nucleotides from each other. Importantly, these small changes can have functional consequences, such as altering transcription factor binding sites embedded within ERV regulatory sequences. To make the problem harder, many of these loci are polymorphic within populations and are likely to differ, for any individual, from those present in the current reference genomes. The review by Pisano et al. [1] in this issue describes these problems in depth and discusses how well current sequencing methods can address them.

Despite the presence of so many ERV copies in each genome, our genomes remain relatively stable. This is due in part to the many redundant host control mechanisms used to regulate ERV activity, for which there is much overlap with mechanisms used to silence other repetitive genomic sequences, such as those derived from transposable elements. A broad overview of these various control mechanisms is nicely reviewed by Geis and Goff [2] and includes: DNA methylation at the ERV loci, repressive transcription factors and co-repressor complexes that block transcription, as well as post-transcriptional silencing mechanisms involving small RNAs. Other reviews in this issue tackle these repressive pathways and how they cooperate further. The SetDB1 complex is one of the major repressive mechanisms used to block ERV transcription via deposition of silent H3K9me3 marks, as outlined in detail by Fukuda and Shinkai [3]. Other epigenetic factors that cooperate with the SETDB1 complex include the well-known TRIM28/KAP1 protein and its associated KRAB zinc finger proteins; Margalit et al. [4] present new evidence demonstrating a role for additional TRIM family members, TRIM24 and TRIM33, which cooperate with TRIM28/KAP1 to regulate ERV expression in embryonic stem cells. Finally, Cullen and Schorn [5] look at post-transcriptional regulatory mechanisms and review the roles of tRNA-derived small RNA fragments (tRFs) in blocking ERV replication. In contrast to these studies that elaborate on the complexes that regulate ERVs, Enriquez-Gasca et al. [6] describe the many ways that ERV derived regulatory elements can turn the tables to regulate host gene expression.

Two articles in this issue highlight the importance of taking an evolutionary view of how ERV sequences interact with their hosts, in order to gain insight into how control mechanisms have arisen and continue to evolve. In particular, many mouse ERV loci are still active, such that ERV sequences vary both within mouse populations and across closely related mouse strains. Rebollo et al. [7] present a masterful study identifying a potential master copy of the most active known mouse ERV, intracisternal A-type particles (IAPs). Using a variety of genomic assays, they identify a single intact locus that may be the source of many new ERV insertions in the C3H strain of mice, and which appears to be present in many other mouse strains as well. Identifying these source elements, and the genetic strains in which they are active, provides a crucial background for understanding how, and in what context, ERVs evade host control mechanisms. To complement this study, Elmer and Ferguson-Smith [8] provide a timely review of how ERV control mechanisms differ across mouse strains as host genomes evolve to fight back particularly against active ERVs.

Despite the many redundant control systems designed to silence ERV activity, it is known that silencing of ERVs is imperfect, such that ERV transcriptional activity can occur in both germline and somatic cells. This has led to the hypothesis that unregulated ERV activity might contribute to human disease. To explore this idea in the context of a specific disease, Kolbe et al. [9] present a new study demonstrating that HERV-H sequences are highly expressed in samples from patients with head and neck cancer and may serve as biomarkers for disease progression. In another cancer context, Singh et al. [10] present results showing that HERV-K sequences are bound by the melanocyte lineage transcription factor MITF, and are highly expressed in a subset of melanomas. These two studies add to a wealth of evidence that ERV activity plays a role in cancer development, progression, and treatment, as reviewed by Curty et al. [11] in this issue. Neurodegenerative diseases represent an additional aging-associated context in which activation of ERVs has been implicated in disease onset and progression. In particular, multiple sclerosis was the first neurodegenerative disease for which a connection to HERV-W activity was suggested, with the evidence for HERV-W contributions to MS reviewed by Tarlinton et al. [12].

In summary, this set of papers lays out the state of our knowledge to date on ERV interactions with their host genomes, responses of the host to block ERV activity, and diseases that may involve aberrant de-regulation of ERV activity. Yet, there are still numerous unanswered questions about how ERVs impact, and interact with, their hosts. We anticipate many new and surprising discoveries in this fast-moving field of ERV biology that will likely open up new avenues of research and shed light on how ERVs are linked to health and disease.
